# Integrated Transcriptomics and Targeted Metabolomics Approaches: Comparative Analysis of the Ileum in Neonatal Piglets with Different Birth Weight

**DOI:** 10.3390/ani16020213

**Published:** 2026-01-11

**Authors:** Hyunseo Lee, Gyuseong Kim, Wonvin Choi, Minju Kim

**Affiliations:** 1 School of Animal Life Convergence Science, Hankyong National University, Anseong 17579, Republic of Korea; gustjekek@naver.com (H.L.); rlarbtjd0808@naver.com (G.K.); dnjs3473@gmail.com (W.C.); 2 Institute of Applied Humanimal Science, Hankyong National University, Anseong 17579, Republic of Korea

**Keywords:** neonatal piglets, birth weight, transcriptomics, metabolomics, RNA-seq

## Abstract

This study was conducted to elucidate the molecular and metabolic differences in ileal development according to birth weight in neonatal piglets. A total of 126 neonatal piglets born from Yorkshire × Landrace × Duroc crossbred sows were used, and the top 5% (H group, 1.77 ± 0.02 kg) and bottom 5% (L group, 0.72 ± 0.03 kg) of birth weights were selected for analysis. Ileal tissues were collected for transcriptomic (RNA-seq) and targeted metabolomic (GC–MS) analyses, and selected genes were validated using RT-qPCR. A total of 112 differentially expressed genes (DEGs) were identified, among which RFC3, PCNA, MCM3, MCM10, AURKA, AURKB, CCNB2, CCNA2, CCNF, and SI were significantly upregulated in the H group (*p* < 0.05). These genes were mainly involved in pathways related to DNA replication, cell division, and nutrient digestion and absorption. In addition, metabolomic analysis revealed that pyruvic acid concentrations were significantly higher in the H group (*p* < 0.05), indicating the activation of energy metabolic pathways. These results indicate that high-birth-weight piglets possess a genetic foundation for enhanced cellular proliferation and energy metabolism, and they further highlight potential molecular targets for improving growth performance and intestinal development in low-birth-weight piglets.

## Simple Summary

Birth weight is a key determinant of intestinal development and subsequent growth performance in pigs, but the molecular mechanisms remain unclear. We investigated ileal differences between high- and low-birth-weight piglets using integrated transcriptomic and targeted metabolomic analyses. High-birth-weight piglets exhibited upregulated expression of genes related to DNA replication, cell division, and energy metabolism, accompanied by higher pyruvic acid concentrations. These findings suggest that birth weight is associated with molecular indicators involved in cellular proliferation and energy metabolism, and that these molecular and metabolic characteristics may be related to intestinal function, physiological adaptability, and growth potential in neonatal piglets.

## 1. Introduction

Continuous genetic selection in the swine industry has resulted in modern sows being selected for high prolificacy, with substantially larger litter sizes compared with previous generations [1,2]. While this improvement has enhanced litter size, it has also introduced new challenges, particularly a decline in average neonatal weight and reduced uniformity among piglets [3,4]. Larger litters frequently result in limited uterine space and impaired placental function, which in turn increase the incidence of intrauterine growth restriction (IUGR) piglets [5,6]. IUGR piglets are characterized by low birth weight and immature organ development, resulting in reduced vitality, limited colostrum intake, weakened immunity, and consequently higher mortality rates [7,8]. Specifically, previous studies have indicated that low birth weight piglets are closely associated with compromised intestinal morphology, such as reduced villus height and increased apoptotic signaling, which weakens the gut barrier function [9,10]. Furthermore, their underdeveloped gastrointestinal tract compromises nutrient absorption efficiency, leading to growth retardation and elevated pre- and post-weaning mortality, thereby causing significant economic losses for swine producers [11,12]. Indeed, lighter piglets have been reported to reach market weight later and to exhibit reduced carcass yields [13]. Thus, neonatal body mass and gut maturation are critical determinants of survival and overall growth performance [14]. Therefore, establishing a comprehensive baseline of the molecular and metabolic mechanisms underlying impaired gut development is crucial, as it provides the essential scientific foundation needed to design effective intervention strategies for compromised neonates in the future [15,16].

In particular, the structural and functional readiness of the intestine immediately after birth is closely linked to nutrient uptake and immune competence, and smaller piglets generally exhibit delayed physiological progress, placing them at a disadvantage in terms of performance [17]. Nevertheless, the molecular pathways and metabolic processes through which early body weight affects intestinal function remain insufficiently understood [10,18]. To address this knowledge gap, the present study integrates transcriptomic and metabolomic analyses to elucidate the molecular and metabolic mechanisms linking birth weight to intestinal development. Consequently, fundamental research is needed to establish strategies that promote within-litter uniformity and improve the survival prospects of disadvantaged neonates.

In recent years, omics-based technologies have emerged as powerful tools for elucidating complex biological processes by integrating multi-dimensional datasets, including genomics, proteomics, and metabolomics [19,20]. Therefore, the present study aims to investigate the molecular mechanisms underlying gut development in piglets with varying birth weights, focusing on how these differences affect survival. The findings are expected to provide a scientific foundation that contributes to improved survival of growth-restricted neonates and ultimately enhance swine production systems.

## 2. Materials and Methods

This study was conducted at the research facilities of Hankyong National University, and was approved by the Institutional Animal Care and Use Committee (IACUC) of Hankyong National University (Approval No.: 2024-1).

### 2.1. Animals and Treatments

A total of 126 neonatal piglets with an average birth weight of 1.17 ± 0.02 kg were obtained from 12 multiparous sows (Yorkshire × Landrace × Duroc crossbreed) housed at the same commercial farm, with litter sizes ranging from 9 to 12 piglets. All sows were inseminated using semen supplied by the same artificial insemination company. Body weights were measured immediately after birth, and piglets were assigned to two groups based on birth weight percentiles: the high-birth-weight group (H; top 5%, *n* = 6) and the low-birth-weight group (L; bottom 5%, *n* = 6) in order to ensure a clear distinction in birth weight between groups. Within each group, male and female piglets were selected at equal ratios to minimized potential sex-related effects. The characteristics of neonatal piglets, litters, and the piglets selected for sampling are presented in Supplementary Table S1.

### 2.2. Sample Collection

Sampling of the ileum of the neonatal piglets was performed immediately after body weight measurement. All animal procedures were conducted in accordance with the guidelines approved by the IACUC. Piglets were humanely euthanized in accordance with approved institutional protocols, followed by tissue collection through dissection. Sections of the ileum were excised 5 cm from the junction with the cecum to obtain small intestine samples. The samples were then washed with phosphate-buffered saline (PBS) and immediately flash-frozen in liquid nitrogen and stored at −80 °C until analysis.

### 2.3. RNA Extraction and RNA-Seq

Five ileal samples from each group (H and L) were selected for transcriptomic analysis. Total RNA was extracted from approximately 100 mg of ileal tissue using 1 mL of TRIzol^Ⓡ^ reagent (Thermo Fisher Scientific, Waltham, MA, USA). The extraction process was performed by grinding the frozen ileal samples using a SHG-15D homogenizer (DAIHAN^Ⓡ^, Wonju-si, Gangwon-do, Republic of Korea). The concentration of total RNA was quantified using a Nanodrop F-3100 (Life Real, Hangzhou, China), and RNA quality and integrity were assessed using an Agilent 4200 TapeStation (Agilent, Santa Clara, CA, USA). Only samples with a RNA integrity number (RIN) ≥ 7.0 were used for subsequent library construction. Thereafter, mRNA was isolated using the Poly(A) RNA Selection Kit (Lexogen, Vienna, Austria), and libraries were constructed from cDNA using the CORALL RNA-Seq V2 Library Prep Kit (Lexogen, Vienna, Austria). Poly(A)-based mRNA enrichment and paired-end sequencing were employed to enhance transcript detection efficiency, including moderately and lowly expressed transcripts. Sequencing was performed on a NovaSeq 6000 (Illumina Inc., San Diego, CA, USA) with 100 bp reads, generating approximately 4 Gb of paired-end sequencing data per sample, which is generally considered sufficient for comprehensive mRNA-seq analysis, including the detection of low-abundance transcripts. For quality control, raw sequencing reads were processed using FASTP 1.0 with default parameters, including adapter removal, filtering of low-quality reads, and automatic application of minimum read length and base quality thresholds. After trimming, only high-quality reads were retained and aligned to the reference genome using STAR 2.7.11b, and the resulting mapped reads were used for downstream analyses. Gene expression levels were calculated using the Salmon *1.10.x* tool. Data normalization was performed using counts per million (CPM) to adjust for differences in library size, followed trimmed mean of M-values (TMM) normalization to correct for expression composition bias and increase the reliability of the results.

### 2.4. RNA-Seq Data Analysis

RNA-Seq data were analyzed using ExDEGA (v5.2.1; ebiogen, Seoul, Republic of Korea), a Microsoft Excel-based software for differential gene expression analysis. Differential expression analysis was performed on a total of 27,376 annotated genes based on the Sus scrofa reference genome. DEGs were selected based on the following criteria: fold change ≥ 1.5 and a normalized log_2_ expression value ≥ 4. Differences in gene expression levels between groups were considered statistically significant at an adjusted *p*-value (FDR) < 0.05. Functional annotation of the identified DEGs was performed using the Database for Annotation, Visualization, and Integrated Discovery (DAVID; https://davidbioinformatics.nih.gov/summary.jsp (accessed on 1 October 2025)). Gene ontology (GO) analysis included the classification of DEGs into biological process (BP), cellular component (CC), and molecular function (MF) categories and pathway analysis based on the Kyoto Encyclopedia of Genes and Genomes (KEGG). KEGG pathway mapping was performed using the KEGG Mapper tool (https://www.genome.jp/kegg/mapper/ (accessed on 1 October 2025)). For both DAVID and KEGG analyses, *Sus scrofa* was selected as the reference species for gene symbol annotation. Data visualization was performed using GraphicPlus, an integrated visualization tool within the ExDEGA software. DEG patterns were visualized using the Multi Experiment Viewer (MeV) 4.9.0 software available from SourceForge (https://sourceforge.net/ (accessed on 1 October 2025)).

### 2.5. Selecting Genes and Real-Time qPCR

Genes were selected through functional annotation analysis of DEGs. The upregulated genes in group H were involved in DNA replication, cell division, mitotic cell cycle phase transition, carbohydrate binding, and nutrient metabolism. The downregulated genes in group H were associated with collagen trimer and serine-type endopeptidase complex.

To validate the mRNA-seq data, real-time reverse transcription polymerase chain reaction (RT-qPCR) was performed. RT-qPCR analyses were conducted using ileal samples from the same piglets used for RNA-seq analysis, with five biological replicates per group (H and L). Each sample was analyzed in triplicate to ensure technical reproducibility. Total RNA was extracted from approximately 100 mg of ileal tissue using TRIzol^Ⓡ^ reagent (Thermo Fisher Scientific, Waltham, MA, USA), and genomic DNA contamination was removed using the gDNA eraser premix (Takara Bio Inc., Shiga, Japan). The purified RNA was then reverse-transcribed into cDNA using the PrimeScript™ FAST RT Reagent Kit (RR092A; Takara Bio Inc., Shiga, Japan) according to the manufacturer’s instructions. RT-qPCR was performed using TB Green^®^ Premix Ex Taq™ II FAST qPCR (RR830A; Takara Bio Inc., Shiga, Japan) and the qTOWER3G real-time PCR system (Analytik Jena GmbH, Jena, Germany), following the manufacturer’s protocol. Glyceraldehyde-3-phosphate dehydrogenase (GAPDH) was used as the housekeeping gene for normalization of relative gene expression levels. The primer sequences used for target gene validation are listed in Supplementary Table S2. Relative gene expression levels were calculated using the 2^−ΔΔCt^ method, normalized to GAPDH expression, and subsequently converted to log_2_ fold change values.

### 2.6. GC-MS Analysis

GC-MS analysis of metabolites was conducted according to [21]. Ileal tissue (20 ± 0.05 mg) was homogenized in 700 μL of extraction solvent (acetonitrile:triple-distilled water, 7:3, *v*/*v*; Duksan General Science, Seoul, Republic of Korea) using an SHG-15D homogenizer (DAIHAN^Ⓡ^, Wonju-si, Gangwon-do, Republic of Korea). The homogenate was centrifuged, and the supernatant was collected and dried using a HyperVAC-LITE (Hanil Scientific Inc., Gimpo, Republic of Korea). For derivatization, 50 μL of MTBSTFA + 1% TBDMCS (N-tert-butyldimethylsilyl-N-methyltrifluoroacetamide + 1% tert-butyldimethylchlorosilane; Thermo Fisher Scientific, Waltham, MA, USA) was added, while BSTFA (N,O-bis(trimethylsilyl)trifluoroacetamide; Sigma-Aldrich, St. Louis, MO, USA) was used specifically for isocitric acid. Standard compounds, including pyruvic acid, succinic acid, fumaric acid, γ-aminobutyric acid (GABA), malic acid, glyoxylic acid, cis-aconitic acid, L-glutamine, citric acid, α-ketoglutaric acid, L-glutamic acid, and isocitric acid (all purchased from Sigma-Aldrich, St. Louis, MO, USA), were used to construct calibration curves at concentrations of 0.5, 2.0, 5.0, 10, 25, and 50 μM, with R^2^ values greater than 0.99. GC–MS analysis was performed on a QP2020NX system (Shimadzu Scientific Korea, Seoul, Gangnam, Republic of Korea) equipped with an HP-5ms capillary column (30 m × 0.25 mm × 0.25 μm; Agilent, Santa Clara, CA, USA). Samples (1 μL) were injected in split mode (1:25). The oven program was as follows: initial temperature of 50 °C for 2 min, increased to 150 °C at 20 °C/min, and then to 300 °C at 8 °C/min. High-purity helium (Airkorea, Yeoju-si, Gyeonggi-do, Republic of Korea) was used as the carrier gas at a constant flow rate of 1 mL/min. The ion source and interface temperatures were set at 280 °C, and data were acquired in SIM mode over an *m*/*z* range of 30–650. For the GC–MS data, normalization was performed using probabilistic quotient normalization (PQN), with the H group used as the reference, followed by log_2_ transformation Pareto scaling, MetaboAnalyst 6.0 (https://www.metaboanalyst.ca/ (accessed on 1 October 2025)) to generate heatmaps and principal component analysis (PCA) plots, thereby assessing group distribution and variance.

### 2.7. Correlation Analysis Between Transcriptome and Metabolome

Based on the transcriptomic analysis, a total of 11 DEGs associated with DNA replication, cell division, and mitotic cell cycle phase transition were selected for correlation analysis. Targeted metabolomic analysis was performed using 12 selected metabolites representative of energy metabolism. To evaluate the associations between gene expression levels and metabolite concentration, Spearman’s rank correlation analysis was conducted using individual-level data (*n* = 10). Given the limited sample size, a non-parametric approach was applied. Statistical significance of individual correlations was initially assessed using a threshold of *p* < 0.05. To account for multiple testing, false discovery rate (FDR) correction was applied, and correlations with *p*-value (FDR) < 0.05 were considered additionally significant. Correlation strength was interpreted using predefined thresholds, with |ρ| ≥ 0.50 classified as moderate correlations and |ρ| ≥ 0.80 classified as strong correlations. The results were visualized as a heatmap, with genes ordered according to their functional categories, to explore overall association patterns between gene functional groups and metabolites. All correlation analyses were performed using RStudio software version 2025.09.2.

### 2.8. Statistical Analysis

To evaluate the significance of differences in body weight and RT-qPCR results of the selected neonatal piglets, statistical analyses were conducted using SAS software version 9.4 (SAS Institute Inc., Cary, NC, USA). Because only one piglet per litter was selected for each group, potential litter effects were minimized by study design. Therefore, litter was not included as a random effect in the statistical model. Prior to statistical analysis, data were assessed for normality using the Shapiro–Wilk and for homogeneity of variance using Levene’s test, and no violations of these assumptions were detected. Independent two-sample *t*-tests were applied to compare body weights, RT-qPCR results, and GC–MS data between the two groups (H and L). Statistical significance was considered at *p* < 0.05, while 0.05 ≤ *p* < 0.10 was regarded as a tendency. For transcriptomic analysis, DEGs were identified using appropriate statistical models, and *p*-values were adjusted for multiple testing using the Benjamini–Hochberg FDR. Genes with an adjusted *p*-value (FDR) < 0.05 were considered statistically significant.

## 3. Results

### 3.1. DEGs Between the H and L Group

**Figure 1:**
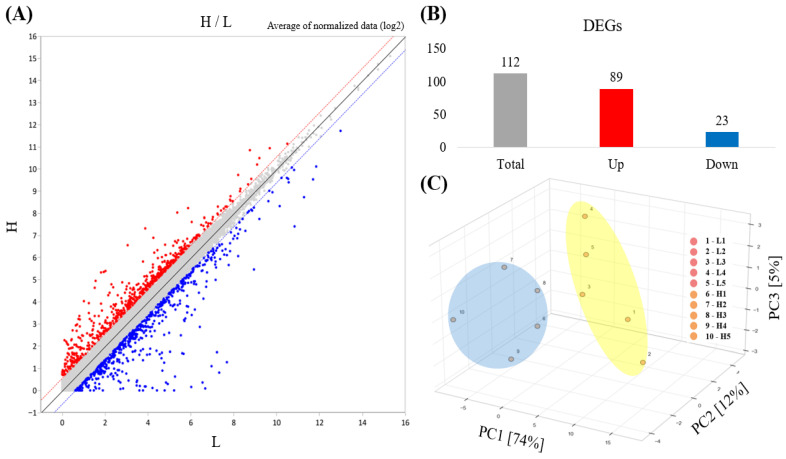
Overview of DEGs in the ileum of piglets between the H and L groups (Fold change ≥ 1.5; normalized expression (log_2_) ≥ 4.0; adjusted *p* < 0.05 (FDR); *n* = 5). (**A**) Scatter plot of DEGs between the H and L groups. Red dots indicate genes upregulated in the H group relative to the L group, while blue dots indicate downregulated genes. Grey dots indicate features that do not meet the predefined threshold for differential expression. (**B**) Number of DEGs identified between the groups. The gray bar represents the total number of DEGs identified between the H and L groups, while the red and blue bars indicate genes upregulated and downregulated in the H group, respectively. (**C**) PCA based on the expression profiles of DEGs in the H and L groups. The H group is represented by the blue ellipse, whereas the L group is represented by the yellow ellipse. The red dots and numbers (1, 2, 3, 4, and 5) represent data from individual neonatal piglet samples in the L group, whereas the orange dots and numbers (6, 7, 8, 9, and 10) represent data from individual neonatal piglet samples in the H group.

**Figure 2:**
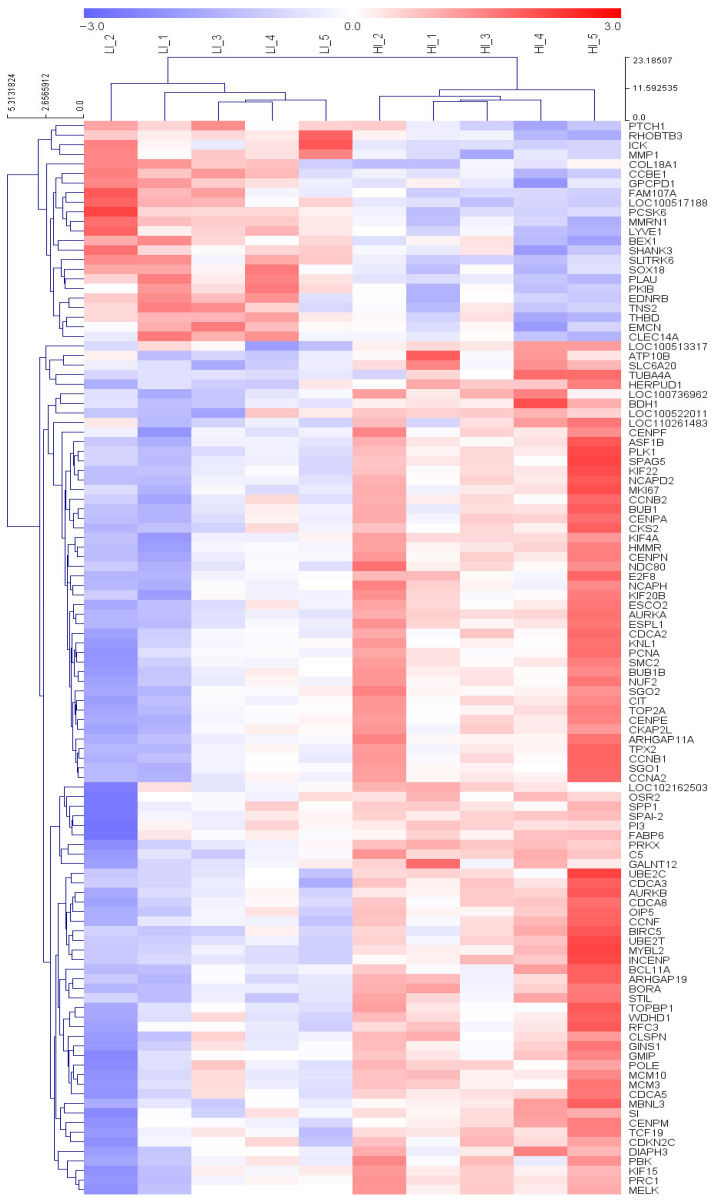
Clustering heatmap of 112 DEGs identified in the ileum of pigs from the H and L groups (*n* = 5). Red indicates upregulated genes, while blue indicates downregulated genes. The expression level of each gene is represented by a color gradient, with deep red indicating higher expression and deep blue indicating lower expression.

In total, 27,376 transcripts were detected by RNA-seq analysis. Based on the criteria of fold change ≥ 1.5, normalized expression (log_2_) ≥ 4, and an adjusted *p*-value (FDR) < 0.05, 112 DEGs were identified between the H and L groups and are listed in (Supplementary Table S3). 89 of these DEGs were upregulated and 23 were downregulated in group H relative to those in group L (*p* < 0.05) (Figure 1B). A scatter plot visualizing the distribution of the upregulated and downregulated DEGs between the H and L groups is shown in (Figure 1A). Principal component analysis (PCA) based on the DEG expression profiles is shown in (Figure 1C), with the first and second principal components (PC1 and PC2) explaining 74% and 12% of the variance, respectively, clearly distinguishing between the H and L groups. Furthermore, a hierarchical clustering heatmap of ileal gene expression patterns revealed distinct transcriptional differences between the two groups (Figure 2).

### 3.2. Functional Annotation Analysis of DEGs

**Figure 3:**
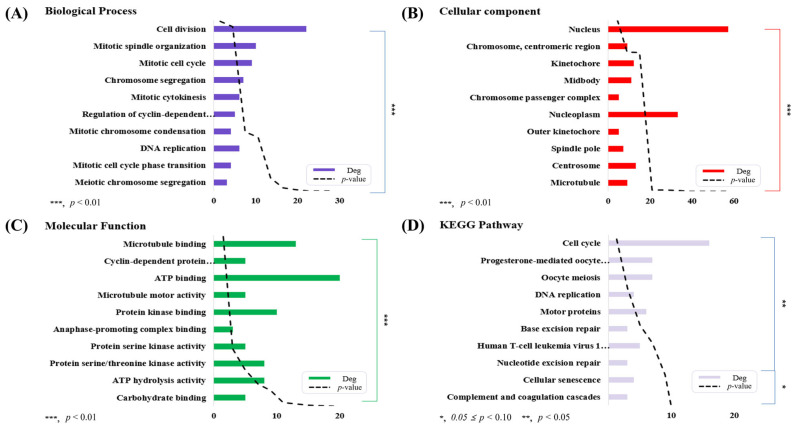
Functional annotation analysis of DEGs between the H and L groups, including BP, CC, MF, and KEGG pathways (*n* = 5). The top 10 GO terms were selected based on *p*-value (indicated by the dashed line), and each bar represents DEGs. In addition, the functional annotations and KEGG pathways indicated by “…” in the figure can be found in Supplementary Tables S4 (Biological Process), S6 (Molecular Function), and S7 (KEGG Pathway). (**A**) Biological Process. (**B**) Cellular Component. (**C**) Molecular Function. (**D**) KEGG Pathway.

Functional annotation of the DEGs between the H and L groups was conducted using DAVID, focusing on the top 10 enriched terms based on *p*-value within the categories of Biological Process (BP), Cellular Component (CC), Molecular Function (MF), and KEGG pathways. The results are shown in (Figure 3A–D). The top 10 BP terms included: cell division, mitotic spindle organization, mitotic cell cycle, chromosome segregation, mitotic cytokinesis, regulation of cyclin-dependent protein serine/threonine kinase activity, mitotic chromosome condensation, DNA replication, mitotic cell cycle phase transition, and meiotic chromosome segregation. The upregulated and downregulated genes associated with each GO term are listed in (Supplementary Table S4).

For CC terms, the top 10 enriched categories were: nucleus, chromosome, centromeric region, kinetochore, midbody, chromosome passenger complex, nucleoplasm, outer kinetochore, spindle pole, centrosome, and microtubule. The DEGs associated with each CC term are listed in (Supplementary Table S5).

The top 10 MF terms included: microtubule binding, cyclin-dependent protein serine/threonine kinase regulator activity, ATP binding, microtubule motor activity, protein kinase binding, anaphase-promoting complex binding, protein serine kinase activity, protein serine/threonine kinase activity, ATP hydrolysis activity, and carbohydrate binding. The associated upregulated and downregulated genes are listed in (Supplementary Table S6).

The top 10 enriched KEGG pathways were: cell cycle, progesterone-mediated oocyte maturation, oocyte meiosis, DNA replication, motor proteins, base excision repair, human T-cell leukemia virus 1 infection, nucleotide excision repair, cellular senescence, and complement and coagulation cascades. The DEGs associated with each KEGG pathway are listed in (Supplementary Table S7).

### 3.3. Quantitative RT-qPCR Validation of DEGs Expression

**Figure 4:**
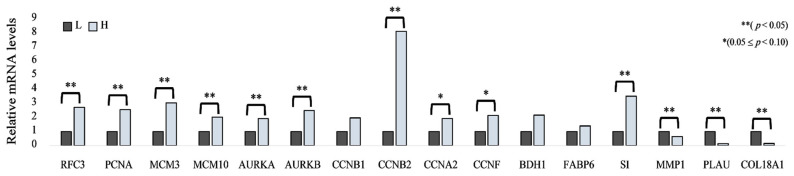
Relative mRNA expression levels of selected genes between neonatal piglets in the H and L groups (*n* = 5). Bars represent mean values, with light gray bars indicating the H group and dark gray bars indicating the L group. ** indicates a significance of *p* < 0.05; * indicates a 0.05 ≤ *p* < 0.10.

The results of RT-qPCR analysis for the selected genes are presented in Figure 4. RT-qPCR validation was performed using ileal samples from five biological replicates per group (H and L). Relative gene expression levels were calculated using the 2^−ΔΔCt^ method and normalized to GAPDH expression. *RFC3*, *PCNA*, *MCM3*, *MCM10*, *AURKA*, *AURKB*, *CCNB2*, and *SI* genes were relatively upregulated in the H group compared to the L group (*p* < 0.05). In addition, *CCNA2* and *CCNF* genes showed a tendency to be upregulated in the H group (0.05 ≤ *p* < 0.10). In contrast, *MMP1*, *PLAU*, and *COL18A1* genes were downregulated in the H group (*p* < 0.05).

### 3.4. Targeted Metabolomics for Energy Metabolism

**Figure 5:**
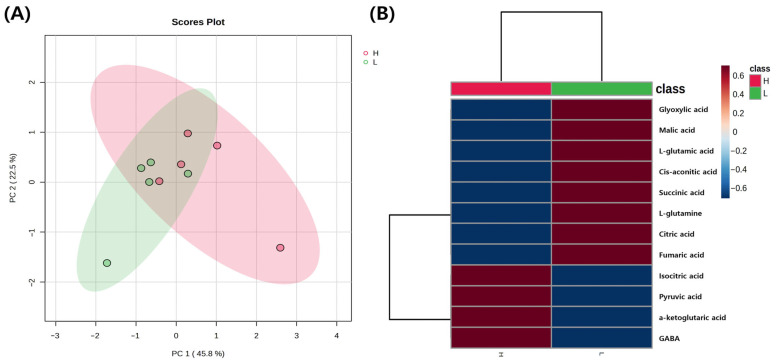
Metabolite analysis results of the H and L groups (*n* = 5). (**A**) PCA results between the H and L groups. The red ellipse represents the H group, while the green ellipse represents the L group. Each ellipse indicates the 95% confidence interval. Red dots represent individual samples from the H group, while green dots represent individual samples from the L group. (**B**) Heatmap between the H and L groups. In the class bar at the top, red indicates the H group and green indicates the L group. For relative metabolite levels, red represents higher metabolite levels, whereas blue represents lower metabolite levels.

**Figure 6:**
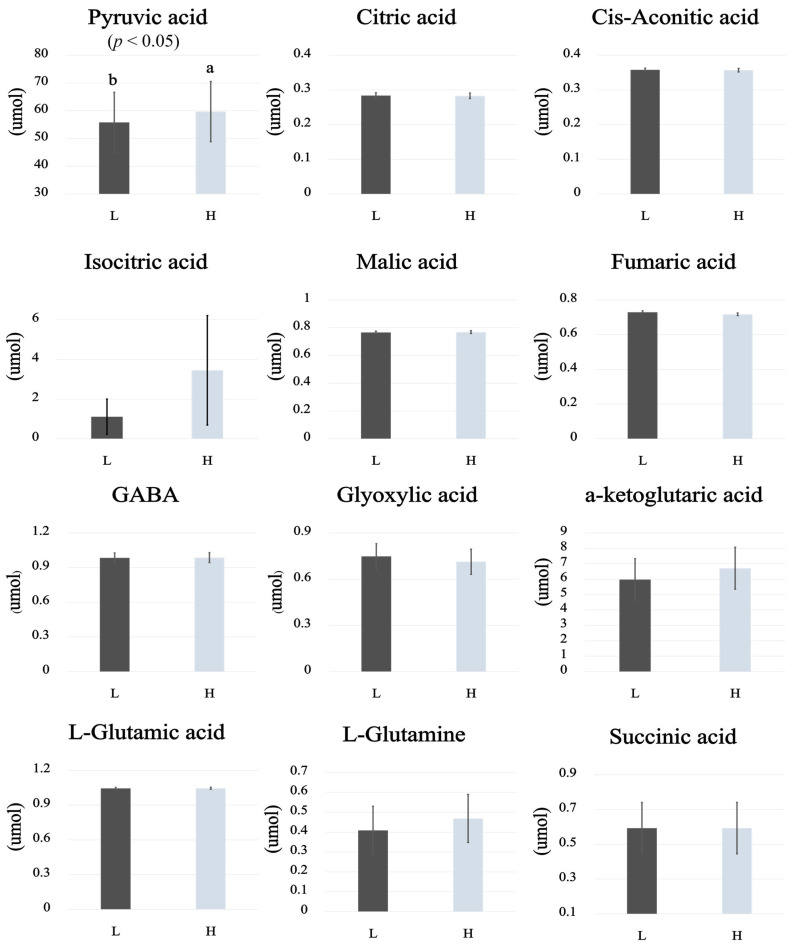
Comparative metabolite profiles between the H and L groups (*n* = 5). Each bar graph represents the relative concentration of individual metabolites, with light gray bars indicating the H group and dark gray bars indicating the L group. Significant differences (*p* < 0.05) are indicated by superscript letters a and b.

The results are presented in Figure 5 and Figure 6. The PCA and heatmap are presented in Figure 5A and Figure 5B, respectively. The PCA explained 68.3% of the total variance, with PC1 accounting for 45.8% and PC2 for 22.5%. When assessed within the 95% confidence interval, no clear separation was observed between the groups. In contrast, the heatmap revealed that the patterns of individual metabolites were distinguishable between the H and L groups. Pyruvic acid was significantly higher in the H group compared to the L group (*p* < 0.05). The other metabolites, including citric acid, cis-aconitic acid, isocitric acid, α-ketoglutaric acid, L-glutamic acid, L-glutamine, glyoxylic acid, GABA, succinic acid, fumaric acid, and malic acid, were not significantly different between the H and L groups (Figure 5).

### 3.5. Correlation Anaylsis Between Transcriptomic and Metabolomic Data

**Figure 7:**
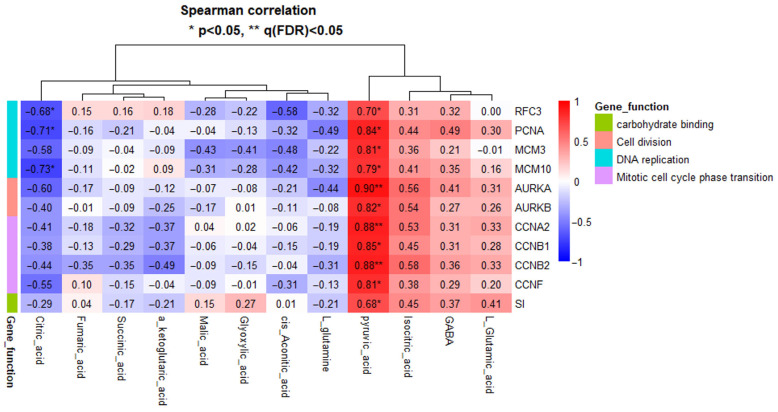
Spearman correlation heatmap between cell cycle–related genes and energy metabolism–related metabolites. Colors indicate the strength and direction of correlations (red, positive; blue, negative). Asterisks denote statistical significance (* *p* < 0.05, ** q(FDR) < 0.05). Genes are grouped according to their functional categories, and hierarchical clustering was applied to both genes and metabolites.

The results of Spearman’s rank correlation analysis between 11 genes associated with DNA replication, cell division, and mitotic cell cycle phase transition and 12 metabolites related to energy metabolism are presented as a heatmap (Figure 7). PCNA, MCM3, AURKA, AURKB, CCNA2, CCNB1, CCNB2, and CCNF showed strong positive correlations with pyruvic acid (ρ > 0.80, *p* < 0.05). In particular, AURKA, CCNA2, and CCNB2 retained significant strong correlations with pyruvic acid even after FDR correction (ρ > 0.80, *p* < 0.05, q < 0.05). In addition, RFC3, MCM10, and SI also exhibited moderate to strong positive correlations with pyruvic acid (ρ > 0.50, *p* < 0.05). In contrast, RFC3, PCNA, and MCM10 showed moderate to strong negative correlations with citric acid (−0.80 ≤ ρ < −0.50). Detailed correlation coefficients and corresponding *p* and q-values for all gene-metabolite pairs are provided in Supplementary Table S8.

## 4. Discussion

### 4.1. DNA Replication

**Figure 8:**
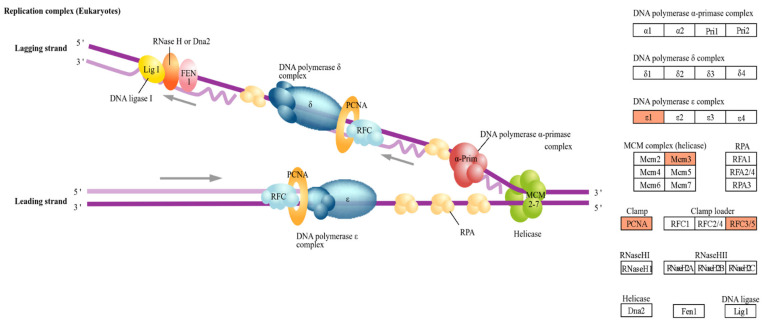
KEGG pathway of DNA replication in the ileum. Genes upregulated in the H group are highlighted in orange.

The small intestine of neonatal piglets undergoes rapid development through continuous epithelial cell turnover, which is essential for intestinal function and regeneration [22]. Accordingly, tight regulation of DNA replication during early cell-cycle phases is critical for sustaining epithelial renewal during neonatal intestinal maturation [23,24]. In particular, accurate DNA replication in early cell-cycle phases is closely associated with subsequent cell division and intestinal development [25]. In this study, DEGs involved in the DNA replication pathway were identified (Figure 8). Increased expression of RFC3 and PCNA has been reported to be associated with the formation of the DNA replication complex and the efficiency of the replication process [26,27]. In pigs and chickens, PCNA expression in the small intestine has been widely used as an indicator of DNA replication and cell proliferation and is associated with intestinal epithelial regeneration [28,29,30]. In pigs, PCNA expression increases during the suckling period but is transiently suppressed by weaning stress [31]. In addition, increased expression of MCM3 and MCM10 in pigs has been associated with the maintenance of genome stability and intestinal epithelial cell proliferation, whereas reduced expression of these genes has been linked to cell-cycle abnormalities and impaired cell proliferation [32,33,34,35]. Taken together, the upregulation of DNA replication–related genes observed in the ileum of high-birth-weight neonatal piglets may reflect molecular features associated with intestinal epithelial proliferation and maturation.

### 4.2. Cell Division and Mitotic Cell Cycle Phase Transition

The genes AURKA and AURKB, which are involved in cell division, were upregulated in the H group. AURKA and AURKB function as key regulatory factors during mitosis and chromosome segregation [36,37]. In pigs, deficiency of AURKA and AURKB has been reported to impair embryonic development through disruptions in spindle formation, microtubule assembly, and chromosome alignment [38,39,40]. In addition, CCNB2, CCNF, and CCNA2, which were also upregulated in the H group, have been reported to be associated with intestinal epithelial cell proliferation and with the regulation of the timing and fidelity of cell division [41,42,43]. Taken together, the upregulation of aurora kinases and cell cycle–related cyclin genes observed in the H group may reflect transcriptional features associated with mitotic progression in the ileal epithelium of high-birth-weight piglets. When considered alongside the upregulation of DNA replication–related genes described in Section 4.1, these findings suggest coordinated transcriptional changes linking DNA synthesis with subsequent cell division. Such coordination is closely associated with epithelial turnover during postnatal intestinal development and may be relevant to differences in intestinal maturation observed between birth-weight groups.

### 4.3. Nutrient Digestion and Absorption

SI functions as a digestive enzyme essential for carbohydrate digestion and energy supply, and its deficiency has been associated with reduced energy absorption and gastrointestinal dysfunction [44,45]. In pigs, SI plays a central role in the hydrolysis of dietary sucrose in the small intestine and is directly involved in carbohydrate digestion and absorption [46]. Ayuso et al. [9] reported that low-birth-weight neonatal piglets exhibited delayed intestinal development accompanied by reduced SI expression, which was associated with impaired postnatal growth. Similarly, Villagómez-Estrada et al. [47] observed lower SI expression in low-birth-weight piglets, consistent with the findings of the present study. From a functional perspective, higher SI expression may reflect transcriptional features associated with carbohydrate digestion and absorption capacity in the intestine. Such expression patterns may be relevant to differences in intestinal metabolic activity during early postnatal development between birth-weight groups.

### 4.4. Energy Metabolism

Metabolomic analysis of the ileum revealed a significantly higher concentration of pyruvic acid in the H group. As the end product of glycolysis, pyruvic acid is a key metabolic intermediate associated with cellular energy metabolism [48]. When considered together with the upregulation of genes related to DNA replication, mitotic progression, and carbohydrate metabolism observed in the H group, the increased pyruvic acid level may reflect differences in intestinal energy metabolic features during early postnatal life. These findings are consistent with previous reports showing higher pyruvic acid levels in heavier piglets compared with low-birth-weight piglets, supporting an association between birth weight and early-life energy metabolism [49].

No significant differences were observed in most other metabolites between the H and L groups, which may reflect the physiological regulation of energy balance and metabolic homeostasis in neonatal piglets immediately after birth [50]. In addition, the limited sample size may have reduced the statistical power to detect subtle differences in additional metabolites.

### 4.5. Association Between Cell Cycle–Related Transcription and Energy Metabolism

In the transcriptomic–metabolomic correlation analysis, pyruvic acid showed positive correlations with the expression of cell cycle–related genes, whereas RFC3, PCNA, and MCM10 exhibited negative correlations with citric acid. These findings suggest that cell cycle–associated transcriptional features may be observed in conjunction with metabolic characteristics related to glycolysis rather than the tricarboxylic acid cycle [51,52,53,54]. In addition, SI showed a positive correlation with pyruvic acid. However, these results are based on correlation analysis with a limited sample size and therefore have limitations in terms of causal inference and functional interpretation.

## 5. Conclusions

In this study, integrated transcriptomic and targeted metabolomic analyses were conducted to explore molecular differences in the ileum of neonatal piglets with different birth weights. The results showed that piglets in the high-birth-weight (H) group exhibited relatively higher expression of genes related to cell division, DNA replication, and energy metabolism, suggesting the presence of differences in transcriptional characteristics associated with intestinal development. In addition, targeted metabolomic analysis revealed a significantly higher concentration of pyruvic acid in the H group, which may reflect differences in energy metabolic features associated with glycolytic processes.

Taken together, these transcriptomic and metabolomic findings indicate that high-birth-weight piglets can be characterized as a group in which molecular features related to intestinal epithelial proliferation and energy metabolism are observed during the early postnatal period. The dataset generated in this study may serve as a foundational resource for exploring candidate genes and metabolic pathways associated with neonatal intestinal development. However, as the present findings are based on observational analyses with a limited sample size, further studies incorporating larger cohorts as well as functional or histological validation are required to clarify the physiological relevance of these molecular features and to establish causal relationships between birth weight and intestinal development.

## Data Availability

Upon reasonable request, the datasets of this study can be available from the corresponding author.

## Supplementary Materials

The following supporting information can be downloaded at: https://www.mdpi.com/article/10.3390/ani16020213/s1. Supplementary Table S1: Characteristics of litters, newborn piglets, and piglets selected for sampling; Supplementary Table S2: Primer sequences of selected genes for RT-qPCR; Supplementary Table S3: Expression levels of differentially expressed genes between the H and L groups; Supplementary Table S4: Functional annotation analysis of differentially expressed genes is H and L groups—Biological process results; Supplementary Table S5: Functional annotation analysis of differentially expressed genes is H and L groups—Cellular component results; Supplementary Table S6. Functional annotation analysis of differentially expressed genes is H and L groups—Molecular function results; Supplementary Table S7: Functional annotation analysis of differentially expressed genes is H and L groups—KEGG pathway results; Supplementary Table S8: Spearman’s rank correlation coefficients and associated *p* and q-values between genes and metabolites.
