# Supplementary material for: Integrated Transcriptomics and Targeted Metabolomics Approaches: Comparative Analysis of the Ileum in Neonatal Piglets with Different Birth Weight
Source: Animals (Basel). 2026 Jan 11;16(2):213. doi: 10.3390/ani16020213 (PMC12837496; doi:10.3390/ani16020213)
Supplement: Supplementary file 1 [file animals-16-00213-s001.zip › animals-3982921-supplementary.pdf]

animals

Supplementary Table S1. Characteristics of litters, newborn piglets, and piglets selected for sampling

**Sows**					
Total number of sows	12				
Average number born alive	10.5				
Average parity	3.16				
Parity 2	2				
Number of sows *Parity 3*	6				
Parity 4	4				
Neonatal piglets					
Total number of piglets	*126*				
Average birth weight (kg)	1.17 ± 0.02				
% male pigs	53.17				
% female pigs	46.83				
**Birth weight of neonatal piglets**					
Range of birth weight	*< 0.8 kg*	*0.8 – 0.99 kg*	*1.0 – 1.49 kg*	*1.5 – 1.65 kg*	*1.7 – 1.85 kg*
Number of piglets (%)	7 (5.56)	28 (22.22)	72 (57.14)	12 (9.52)	7 (5.56)
Average birth weight (kg)	0.70 kg ± 0.03	0.93 kg ± 0.01	1.19 kg ± 0.02	1.57 kg ± 0.02	1.78 kg ± 0.02
Selected piglets for sampling					
Group	L	H	p-value		
Number of piglets	6	6	-		
Average birth weight (kg)	0.72^{b} ± 0.03	1.77^{a} ± 0.02	< 0.001		

Supplementary Table S2. Primer sequences of selected genes for RT-qPCR

Name	Sequence forward (5'-3')	Sequence reverse (5'-3')	Product size	GenBank accesstion number
RFC3	ACTGTATGGTGTTGGCGTGG	CAAGTTGCTGTGATTGCGCC	191	NM_001190192.1
PCNA	GCCACTCCACTCTCTCCTACA	GGCATCACCGAAGCAGTTCTC	194	NM_001291925.1
MCM3	GCGCAGAGAGACTACCTGGA	CTTTAAGGCCCGCTGGAAGG	201	XM_001924813.6
MCM10	CTTGACGTCCCAACTCTGCC	GGGTCCCTGCACTCTGATCT	172	XM_021064865.1
AURKA	TGGATCTCTGGAGCTTGGGG	TGAGATGAGGTCCCTGGCTC	155	XM_005673034.3
AURKB	CATGAGCCGCTCCAATACCC	GACTTTGAGCGCCACGATGA	199	NM_213919.2
CCNB1	GCTCTGCTTCTCCCCTCTGA	GCAGTTCTTGGCCTCAGTCC	181	NM_001170768.1
CCNB2	GGAAAACCCTCAGCTCTGCA	GATGGCCACGCACATGTACA	205	NM_001114282.1
CCNF	GAGAAACAGAACCAGGCCCG	GCGAAGTCCCTGAGTTTCCG	152	XM_003124740.6
CCNA2	GGAGCAGTGATGTTGGGCAG	TGAGTTCCCGGCCTTCAGTA	183	NM_001177926.1
BDH1	CAACTTCATCGTCGCCACCA	TGGGGTGGTAGCGAGTGTAG	236	XM_021070091.1
SI	ACAGAGCCTTACCCTGCCTT	CTTGCCCACCATTCTGCTGT	215	XM_021069748.1
FABP6	CTCATCCTTCGGCTCCTGGA	CCGATGGTGAACGTGTTGGT	240	NM_214215.2
PLAU	CACGTACCATGCCCACAGAC	ATAAGCAGGCCGGTGACTCT	178	NM_213945.1
MMP1	CGTGTGGCTGCTCATGAACT	CACTTGGCTGAACGGGGTTT	172	NM_001166229.1
COL18A1	GCAGAGACGTCCTTCAGCAC	CTACTTGGAGGCGGAGGTCA	242	XM_021071536.1
GAPDH	GGGTCATCATCTCTGCCCCT	GGTCATGAGTCCCTCCACGA	176	NM_001206359.1

Supplementary Table S3. Expression levels of differentially expressed genes between the H and L groups

Gene symbol	[1)]FC(H/L)	p-value	Gene symbol	[1)]FC(H/L)	p-value	Gene symbol	[1)]FC(H/L)	p-value
Up DEGs								
LOC100513317	3.257	0.034	*CDCA8*	1.753	0.013	*C5*	1.918	0.001
MBNL3	1.621	0.022	*BUB1*	1.670	0.046	*STIL*	1.713	0.003
AURKA	1.778	0.002	*PLK1*	1.669	0.019	*PRC1*	1.556	0.009
LOC100736962	2.361	0.004	*DIAPH3*	1.915	0.026	*ESPL1*	1.984	0.009
UBE2C	1.550	0.032	*TOP2A*	1.759	0.027	*KIF15*	1.506	0.028
MYBL2	2.036	0.036	*SPAG5*	1.628	0.029	*CDCA3*	1.587	0.011
TPX2	1.602	0.041	*LOC100522011*	1.540	0.023	*NCAPD2*	1.569	0.014
PCNA	1.614	0.043	*NCAPH*	1.651	0.027	*BUB1B*	1.635	0.029
ATP10B	1.738	0.036	*KNL1*	1.545	0.049	*CLSPN*	1.567	0.017
HMMR	1.801	0.011	*OIP5*	1.516	0.024	*CENPN*	1.581	0.010
SGO2	1.572	0.042	*PRKX*	1.527	0.002	*LOC102162503*	2.730	0.027
CCNB1	1.587	0.028	*CENPF*	1.510	0.014	*HERPUD1*	1.617	0.005
KIF4A	1.675	0.005	*CCNF*	1.526	0.012	*PBK*	1.503	0.040
GINS1	1.504	0.041	*MCM10*	1.688	0.015	*PI3*	2.013	0.048
CIT	1.711	0.018	*CENPE*	1.689	0.022	*FABP6*	2.894	0.043
POLE	1.741	0.024	*CKAP2L*	1.701	0.031	*INCENP*	1.560	0.013
ESCO2	1.76	0.021	*MCM3*	1.624	0.036	*NDC80*	1.677	0.010
CDCA2	1.567	0.023	*KIF22*	1.624	0.025	*RFC3*	1.543	0.044
BDH1	1.561	0.014	*WDHD1*	1.571	0.038	*GALNT12*	1.553	0.033
SI	3.710	0.038	*SMC2*	1.682	0.020	*CDCA5*	1.527	0.027
SLC6A20	1.742	0.005	*TUBA4A*	1.565	0.045	*LOC110261483*	1.643	0.037
SGO1	1.703	0.041	*CCNB2*	1.521	0.034	*TCF19*	1.523	0.019
TOPBP1	1.723	0.040	*E2F8*	1.714	0.031	*NUF2*	1.512	0.024
AURKB	1.684	0.027	*BORA*	1.608	0.004	*MKI67*	1.691	0.038
ARHGAP19	1.505	0.013	*GMIP*	1.557	0.032	*KIF20B*	1.533	0.028
BIRC5	1.581	0.027	*CENPA*	1.540	0.013	*CDKN2C*	1.513	0.031
UBE2T	1.743	0.022	*SPAI-2*	4.251	0.006	*CKS2*	1.565	0.039
CENPM	1.522	0.022	*BCL11A*	1.572	0.036	*CCNA2*	1.718	0.026
SPP1	1.744	0.050	*ASF1B*	1.737	0.022	*MELK*	1.678	0.014
OSR2	1.831	0.036	*ARHGAP11A*	1.514	0.023			
Down DEGs								
BEX1	0.659	0.022	*EDNRB*	0.645	0.017	*MMRN1*	0.532	0.004
THBD	0.610	0.008	*PLAU*	0.654	0.002	*LYVE1*	0.614	0.014
COL18A1	0.631	0.029	*SOX18*	0.511	0.012	*MMP1*	0.515	0.006
SLITRK6	0.606	0.002	*PCSK6*	0.453	0.037	*SHANK3*	0.572	0.031
PTCH1	0.649	0.018	*CCBE1*	0.578	0.012	*TNS2*	0.627	0.041
FAM107A	0.542	0.038	*RHOBTB3*	0.655	0.024	*EMCN*	0.645	0.024
ICK	0.614	0.029	*PKIB*	0.663	0.011	*CLEC14A*	0.661	0.032
LOC100517188	0.443	0.007	*GPCPD1*	0.642	0.021			

[1)]FC(H/L): Fold change threshold relative to the H group was set at ≥ 1.5

Supplementary Table S4. Functional annotation analysis of differentially expressed genes in H and L groups – Biological process results.

Gene ontology	Number of DEGs(H/L)		Genes	$-Log_{10}$ (p-value)
	Up	Down		
Cell division (GO:00531301)	22	0	Up : *SPAG5, CDCA3, CDCA5, CCNF, PLK1, CDCA8, KNL1, NCAPH, NDC80, AURKB, AURKA, CCNA2, SGO1, CCNB2, SGO2, TPX2, CCNB1, INCENP, CKS2, BIRC5, NCAPD2, OIP5*<br>Down :	22.603
Mitotic spindle organization (GO:0007052)	10	0	Up : *CENPE, STIL, CCNB1, KIF4A, NUF2, PLK1, KIF22, AURKB, NDC80, AURKA*<br>Down :	11.082
Mitotic cell cycle (GO:0000278)	9	0	Up : *WDHD1, CENPE, CENPF, PLK1, NCAPD2, MYBL2, POLE, TUBA4A, AURKA*<br>Down :	7.24
Chromosome segregation (GO:0007059)	7	0	Up : *TOP2A, SGO2, DIAPH3, INCENP, BIRC5, OIP5, CENPN*<br>Down :	5.894
Mitotic cytokinesis (GO:0000281)	6	0	Up : *INCENP, KIF4A, PLK1, BIRC5, CENPA, CIT*<br>Down :	5.476
Regulation of cyclin-dependent protein serine/threonine kinase activity (GO:0000079)	5	0	Up : *CCNA2, CCNB2, CCNB1, CDKN2C, CCNF*<br>Down :	4.858
Mitotic chromosome condensation (GO:0007076)	4	0	Up : *CDCA5, NCAPD2, NCAPH, SMC2*<br>Down :	4.247
DNA replication (GO:0006260)	6	0	Up : *GINS1, RFC3, PCNA, MCM3, MCM10, POLE*<br>Down :	4.204
Mitotic cell cycle phase transition (GO:0044772)	4	0	Up : *CCNA2, CCNB2, CCNB1, CCNF*<br>Down :	3.761
Meiotic chromosome segregation (GO:0045132)	3	0	Up : *SGO1, NUF2, SMC2*<br>Down :	3.746

Supplementary Table S5. Functional annotation analysis of differentially expressed genes in H and L groups – Cellular component results

Gene ontology	Number of DEGs(H/L)		Genes	-Log10 (p-value)
	Up	Down		
Nucleus (GO:0005634)	52	5	Up : *ARHGAP11A, TOP2A, CCNF, MCM10, MKI67, SMC2, NUF2, PBK, MYBL2, OIP5, TOPBP1, POLE, TCF19, WDHD1, OSR2, RFC3, BORA, ESCO2, KNL1, CCNA2, SGO1, ESPL1, INCENP, MCM3, BIRC5, KIF20B, ASF1B, PCNA, CDCA2, CDCA5, CDCA8, HMMR, CENPA, AURKB, CCNB2, CCNB1, CLSPN, MBNL3, E2F8, CDKN2C, BCL11A, PLK1, NDC80, TPX2, CENPE, CENPF, DIAPH3, FABP6, UBE2T, CENPM, CENPN, NCAPD2*<br>Down : *FAM107A, SOX18, BEX1, RHOBTB3, PKIB*	10.857
Chromosome, centromeric region (GO:0000775)	9	0	Up : *TOP2A, SGO1, SGO2, CENPF, BIRC5, CDCA8, OIP5, CENPN, AURKB*<br>Down :	10.475
Kinetochore (GO:0000776)	12	0	Up : *SGO1, SGO2, CENPE, SPAG5, INCENP, NUF2, PLK1, BIRC5, BUB1B, BUB1, AURKB, AURKA*<br>Down :	10.461
Midbody (GO:0030496)	10	1	Up : *CENPE, CENPF, INCENP, PRC1, KIF4A, PLK1, BIRC5, CDCA8, KIF20B, AURKB*<br>Down : *PTCH1*	8.313
Chromosome passenger complex (GO:0032133)	5	0	Up : *INCENP, BIRC5, CDCA8, AURKB, AURKA*<br>Down :	7.072
Nucleoplasm (GO:0005654)	33	0	Up : *TOP2A, CDCA2, CDCA5, CDCA8, MCM10, PRKX, CENPA, NCAPH, AURKB, SMC2, NUF2, MYBL2, CLSPN, MBNL3, POLE, BUB1, E2F8, WDHD1, STIL, BCL11A, GMIP, NDC80, CCNA2, SGO1, TPX2, CENPF, PRC1, KIF4A, UBE2T, CENPN, NCAPD2, KIF20B, ASF1B*<br>Down :	6.882
Outer kinetochore (GO:0000940)	5	0	Up : *CENPF, CCNB1, PLK1, BUB1B, BUB1*<br>Down :	6.852
Spindle pole (GO:0000922)	7	0	Up : *SGO1, TPX2, CENPF, CCNB1, DIAPH3, PLK1, AURKA*<br>Down :	4.768
Centrosome (GO:0005813)	13	0	Up : *STIL, CKAP2L, CCNF, PLK1, HMMR, NDC80, AURKA, SGO1, CCNB2, CCNB1, ESPL1, MCM3, KIF20B*<br>Down :	4.766
Microtubule (GO:0005874)	9	0	Up : *TPX2, CENPE, INCENP, BIRC5, KIF22, KIF20B, TUBA4A, AURKA, KIF15*<br>Down :	4.653

Supplementary Table S6. Functional annotation analysis of differentially expressed genes in H and L groups – Molecular function results

Gene ontology	Number of DEGs(H/L)		Gene	-Log10 (p-value)
	Up	Down		
Microtubule binding (GO:0008017)	13	0	Up : *SPAG5, PLK1, KIF22, NDC80, KIF15, CENPE, CENPF, DIAPH3, PRC1, KIF4A, NUF2, BIRC5, KIF20B*<br>Down :	8.226
Cyclin-dependent protein serine/threonine kinase regulator activity (GO:0016538)	5	0	Up : *CCNA2, CCNB2, CCNB1, CCNF, CKS2*<br>Down :	4.647
ATP binding (GO:0005524)	20	1	Up : *TOP2A, UBE2C, PLK1, ATP10B, BUB1B, PRKX, KIF22, AURKB, AURKA, SMC2, CIT, KIF15, CENPE, MELK, KIF4A, UBE2T, PBK, MCM3, KIF20B, BUB1*<br>Down : *RHOBTB3*	4.454
Microtubule motor activity (GO:0003777)	5	0	Up : *CENPE, KIF4A, KIF22, KIF20B, KIF15*<br>Down :	4.014
Protein kinase binding (GO:0019901)	10	0	Up : *CCNA2, TPX2, CCNB1, BORA, CDKN2C, PRC1, PLK1, CKS2, TUBA4A, CIT*<br>Down :	3.782
Anaphase-promoting complex binding (GO:0010997)	3	0	Up : *CCNF, PLK1, CLSPN*<br>Down :	3.13
Protein serine kinase activity (GO:0106310)	5	0	Up : *MELK, PLK1, AURKB, CIT, AURKA*<br>Down :	2.988
Protein serine/threonine kinase activity (GO:0004674)	8	0	Up : *MELK, PLK1, PBK, PRKX, BUB1, AURKB, CIT, AURKA*<br>Down :	2.77
ATP hydrolysis activity (GO:0016887)	7	1	Up : *RFC3, MCM3, ATP10B, KIF22, KIF20B, KIF15, SMC2*<br>Down : *RHOBTB3*	2.571
Carbohydrate binding (GO:0030246)	2	3	Up : *GALNT12, SI*<br>Down : *FAM107A, CLEC14A, GPCPD1*	2.013

Supplementary Table S7. Functional annotation analysis of differentially expressed genes in H and L groups – KEGG pathway results

Gene ontology	Number of DEGs(H/L)		Gene	$-Log_{10}$ (p-value)
	Up	Down		
Cell cycle (ssc04110)	16	0	Up : *PCNA, CDKN2C, CDCA5, PLK1, BUB1B, ESCO2, KNL1, NDC80, AURKB, CCNA2, SGO1, CCNB2, CCNB1, ESPL1, MCM3, BUB1*<br>Down :	14.745
Progesterone-mediated oocyte maturation (ssc04914)	7	0	Up : *CCNA2, CCNB2, CCNB1, PLK1, KIF22, BUB1, AURKA*<br>Down :	5.009
Oocyte meiosis (ssc04114)	7	0	Up : *SGO1, CCNB2, CCNB1, ESPL1, PLK1, BUB1, AURKA*<br>Down :	4.350
DNA replication (ssc03030)	4	0	Up : *RFC3, PCNA, MCM3, POLE*<br>Down :	3.051
Motor proteins (ssc04814)	6	0	Up : *CENPE, KIF4A, KIF22, KIF20B, TUBA4A, KIF15*<br>Down :	2.478
Base excision repair (ssc03410)	3	0	Up : *RFC3, PCNA, POLE*<br>Down :	1.621
Human T-cell leukemia virus 1 infection (ssc05166)	5	0	Up : *CCNA2, CCNB2, CDKN2C, ESPL1, BUB1B*<br>Down :	1.469
Nucleotide excision repair (ssc03420)	3	0	Up : *RFC3, PCNA, POLE*<br>Down :	1.400
Cellular senescence (ssc04218)	4	0	Up : *CCNA2, CCNB2, CCNB1, MYBL2*<br>Down :	1.288
Complement and coagulation cascades (ssc04610)	1	2	Up : *C5*<br>Down : *THBD, PLAU*	1.151

Supplementary Table S8. Spearman's rank correlation coefficients and associated p and q-values between genes and metabolites

	Gene_function	Gene	Metabolite	rho	p-value	q-value
1	DNA replication	RFC3	Pyruvic acid	0.6970	0.0251	0.2761
2	DNA replication	RFC3	Succinic acid	0.1581	0.6628	0.9362
3	DNA replication	RFC3	Fumaric acid	0.1463	0.6866	0.9362
4	DNA replication	RFC3	GABA	0.3161	0.3736	0.8175
5	DNA replication	RFC3	Malic acid	-0.2814	0.4310	0.8245
6	DNA replication	RFC3	Glyoxylic acid	-0.2242	0.5334	0.9235
7	DNA replication	RFC3	Cis-Aconitic acid	-0.5829	0.0770	0.5979
8	DNA replication	RFC3	L-glutamine	-0.3212	0.3655	0.8175
9	DNA replication	RFC3	Citric acid	-0.6770	0.0315	0.2973
10	DNA replication	RFC3	A ketoglutaric acid	0.1758	0.6272	0.9362
11	DNA replication	RFC3	L-Glutamic acid	0.0000	1.0000	1.0000
12	DNA replication	RFC3	Isocitric acid	0.3091	0.3848	0.8175
13	DNA replication	PCNA	Pyruvic acid	0.8424	0.0022	0.0586
14	DNA replication	PCNA	Succinic acid	-0.2067	0.5667	0.9235
15	DNA replication	PCNA	Fumaric acid	-0.1585	0.6618	0.9362
16	DNA replication	PCNA	GABA	0.4863	0.1541	0.8135
17	DNA replication	PCNA	Malic acid	-0.0428	0.9065	0.9797
18	DNA replication	PCNA	Glyoxylic acid	-0.1273	0.7261	0.9584
19	DNA replication	PCNA	Cis-Aconitic acid	-0.3168	0.3725	0.8175
20	DNA replication	PCNA	L-glutamine	-0.4909	0.1497	0.8135
21	DNA replication	PCNA	Citric acid	-0.7078	0.0220	0.2643
22	DNA replication	PCNA	A ketoglutaric acid	-0.0424	0.9074	0.9797
23	DNA replication	PCNA	L-Glutamic acid	0.3016	0.3971	0.8190
24	DNA replication	PCNA	Isocitric acid	0.4424	0.2004	0.8175
25	DNA replication	MCM3	Pyruvic acid	0.8061	0.0049	0.0802
26	DNA replication	MCM3	Succinic acid	-0.0365	0.9203	0.9797
27	DNA replication	MCM3	Fumaric acid	-0.0915	0.8016	0.9681
28	DNA replication	MCM3	GABA	0.2128	0.5551	0.9235
29	DNA replication	MCM3	Malic acid	-0.4282	0.2170	0.8175
30	DNA replication	MCM3	Glyoxylic acid	-0.4061	0.2443	0.8175
31	DNA replication	MCM3	Cis-Aconitic acid	-0.4752	0.1652	0.8175
32	DNA replication	MCM3	L-glutamine	-0.2242	0.5334	0.9235
33	DNA replication	MCM3	Citric acid	-0.5847	0.0759	0.5979
34	DNA replication	MCM3	A ketoglutaric acid	-0.0909	0.8028	0.9681
35	DNA replication	MCM3	L-Glutamic acid	-0.0123	0.9731	0.9943
36	DNA replication	MCM3	Isocitric acid	0.3576	0.3104	0.8175
37	DNA replication	MCM10	Pyruvic acid	0.7939	0.0061	0.0895
38	DNA replication	MCM10	Succinic acid	-0.0182	0.9601	0.9943
39	DNA replication	MCM10	Fumaric acid	-0.1098	0.7628	0.9681

40	DNA replication	MCM10	GABA	0.3526	0.3177	0.8175
41	DNA replication	MCM10	Malic acid	-0.3058	0.3901	0.8175
42	DNA replication	MCM10	Glyoxylic acid	-0.2848	0.4250	0.8245
43	DNA replication	MCM10	Cis-Aconitic acid	-0.4181	0.2292	0.8175
44	DNA replication	MCM10	L-glutamine	-0.3212	0.3655	0.8175
45	DNA replication	MCM10	Citric acid	-0.7262	0.0174	0.2295
46	DNA replication	MCM10	A ketoglutaric acid	0.0909	0.8028	0.9681
47	DNA replication	MCM10	L-Glutamic acid	0.1600	0.6588	0.9362
48	DNA replication	MCM10	Isocitric acid	0.4061	0.2443	0.8175
49	Cell division	AURKA	Pyruvic acid	0.9030	0.0003	0.0358
50	Cell division	AURKA	Succinic acid	-0.0912	0.8022	0.9681
51	Cell division	AURKA	Fumaric acid	-0.1707	0.6372	0.9362
52	Cell division	AURKA	GABA	0.4134	0.2351	0.8175
53	Cell division	AURKA	Malic acid	-0.0673	0.8535	0.9797
54	Cell division	AURKA	Glyoxylic acid	-0.0788	0.8287	0.9681
55	Cell division	AURKA	Cis-Aconitic acid	-0.2091	0.5621	0.9235
56	Cell division	AURKA	L-glutamine	-0.4424	0.2004	0.8175
57	Cell division	AURKA	Citric acid	-0.5970	0.0684	0.5979
58	Cell division	AURKA	A ketoglutaric acid	-0.1152	0.7514	0.9681
59	Cell division	AURKA	L-Glutamic acid	0.3077	0.3870	0.8175
60	Cell division	AURKA	Isocitric acid	0.5636	0.0897	0.6233
61	Cell division	AURKB	Pyruvic acid	0.8182	0.0038	0.0802
62	Cell division	AURKB	Succinic acid	-0.0912	0.8022	0.9681
63	Cell division	AURKB	Fumaric acid	-0.0122	0.9733	0.9943
64	Cell division	AURKB	GABA	0.2675	0.4550	0.8317
65	Cell division	AURKB	Malic acid	-0.1651	0.6484	0.9362
66	Cell division	AURKB	Glyoxylic acid	0.0061	0.9867	0.9943
67	Cell division	AURKB	Cis-Aconitic acid	-0.1140	0.7538	0.9681
68	Cell division	AURKB	L-glutamine	-0.0788	0.8287	0.9681
69	Cell division	AURKB	Citric acid	-0.4000	0.2520	0.8175
70	Cell division	AURKB	A ketoglutaric acid	-0.2485	0.4888	0.8719
71	Cell division	AURKB	L-Glutamic acid	0.2646	0.4600	0.8317
72	Cell division	AURKB	Isocitric acid	0.5394	0.1076	0.6763
73	Mitotic cell cycle phase transition	CCNB1	Pyruvic acid	0.8545	0.0016	0.0540
74	Mitotic cell cycle phase transition	CCNB1	Succinic acid	-0.2918	0.4133	0.8245
75	Mitotic cell cycle phase transition	CCNB1	Fumaric acid	-0.1341	0.7118	0.9584
76	Mitotic cell cycle phase transition	CCNB1	GABA	0.3100	0.3833	0.8175
77	Mitotic cell cycle phase transition	CCNB1	Malic acid	-0.0612	0.8667	0.9797
78	Mitotic cell cycle phase transition	CCNB1	Glyoxylic acid	-0.0424	0.9074	0.9797
79	Mitotic cell cycle phase transition	CCNB1	Cis-Aconitic acid	-0.1457	0.6879	0.9362
80	Mitotic cell cycle phase transition	CCNB1	L-glutamine	-0.1879	0.6032	0.9362
81	Mitotic cell cycle phase transition	CCNB1	Citric acid	-0.3816	0.2766	0.8175

82	Mitotic cell cycle phase transition	CCNB1	A ketoglutaric acid	-0.3697	0.2931	0.8175
83	Mitotic cell cycle phase transition	CCNB1	L-Glutamic acid	0.2770	0.4385	0.8269
84	Mitotic cell cycle phase transition	CCNB1	Isocitric acid	0.4545	0.1869	0.8175
85	Mitotic cell cycle phase transition	CCNB2	Pyruvic acid	0.8788	0.0008	0.0358
86	Mitotic cell cycle phase transition	CCNB2	Succinic acid	-0.3526	0.3177	0.8175
87	Mitotic cell cycle phase transition	CCNB2	Fumaric acid	-0.3537	0.3161	0.8175
88	Mitotic cell cycle phase transition	CCNB2	GABA	0.3647	0.3001	0.8175
89	Mitotic cell cycle phase transition	CCNB2	Malic acid	-0.0856	0.8141	0.9681
90	Mitotic cell cycle phase transition	CCNB2	Glyoxylic acid	-0.1515	0.6761	0.9362
91	Mitotic cell cycle phase transition	CCNB2	Cis-Aconitic acid	-0.0443	0.9032	0.9797
92	Mitotic cell cycle phase transition	CCNB2	L-glutamine	-0.3091	0.3848	0.8175
93	Mitotic cell cycle phase transition	CCNB2	Citric acid	-0.4370	0.2067	0.8175
94	Mitotic cell cycle phase transition	CCNB2	A ketoglutaric acid	-0.4909	0.1497	0.8135
95	Mitotic cell cycle phase transition	CCNB2	L-Glutamic acid	0.3262	0.3577	0.8175
96	Mitotic cell cycle phase transition	CCNB2	Isocitric acid	0.5758	0.0816	0.5981
97	Mitotic cell cycle phase transition	CCNA2	Pyruvic acid	0.8788	0.0008	0.0358
98	Mitotic cell cycle phase transition	CCNA2	Succinic acid	-0.3161	0.3736	0.8175
99	Mitotic cell cycle phase transition	CCNA2	Fumaric acid	-0.1768	0.6250	0.9362
100	Mitotic cell cycle phase transition	CCNA2	GABA	0.3100	0.3833	0.8175
101	Mitotic cell cycle phase transition	CCNA2	Malic acid	0.0367	0.9198	0.9797
102	Mitotic cell cycle phase transition	CCNA2	Glyoxylic acid	0.0182	0.9602	0.9943
103	Mitotic cell cycle phase transition	CCNA2	Cis-Aconitic acid	-0.0570	0.8757	0.9797
104	Mitotic cell cycle phase transition	CCNA2	L-glutamine	-0.1879	0.6032	0.9362
105	Mitotic cell cycle phase transition	CCNA2	Citric acid	-0.4062	0.2441	0.8175
106	Mitotic cell cycle phase transition	CCNA2	A ketoglutaric acid	-0.3697	0.2931	0.8175
107	Mitotic cell cycle phase transition	CCNA2	L-Glutamic acid	0.3323	0.3481	0.8175
108	Mitotic cell cycle phase transition	CCNA2	Isocitric acid	0.5273	0.1173	0.7038
109	Mitotic cell cycle phase transition	CCNF	Pyruvic acid	0.8061	0.0049	0.0802
110	Mitotic cell cycle phase transition	CCNF	Succinic acid	-0.1520	0.6751	0.9362
111	Mitotic cell cycle phase transition	CCNF	Fumaric acid	0.1037	0.7757	0.9681
112	Mitotic cell cycle phase transition	CCNF	GABA	0.2857	0.4236	0.8245
113	Mitotic cell cycle phase transition	CCNF	Malic acid	-0.0856	0.8141	0.9681
114	Mitotic cell cycle phase transition	CCNF	Glyoxylic acid	-0.0061	0.9867	0.9943
115	Mitotic cell cycle phase transition	CCNF	Cis-Aconitic acid	-0.3104	0.3827	0.8175
116	Mitotic cell cycle phase transition	CCNF	L-glutamine	-0.1273	0.7261	0.9584
117	Mitotic cell cycle phase transition	CCNF	Citric acid	-0.5478	0.1012	0.6679
118	Mitotic cell cycle phase transition	CCNF	A ketoglutaric acid	-0.0424	0.9074	0.9797
119	Mitotic cell cycle phase transition	CCNF	L-Glutamic acid	0.1969	0.5855	0.9362
120	Mitotic cell cycle phase transition	CCNF	Isocitric acid	0.3818	0.2763	0.8175
121	carbohydrate binding	SI	Pyruvic acid	0.6848	0.0289	0.2933
122	carbohydrate binding	SI	Succinic acid	-0.1702	0.6383	0.9362
123	carbohydrate binding	SI	Fumaric acid	0.0427	0.9068	0.9797

124	carbohydrate binding	SI	GABA	0.3708	0.2915	0.8175
125	carbohydrate binding	SI	Malic acid	0.1529	0.6732	0.9362
126	carbohydrate binding	SI	Glyoxylic acid	0.2727	0.4458	0.8289
127	carbohydrate binding	SI	Cis-Aconitic acid	0.0127	0.9723	0.9943
128	carbohydrate binding	SI	L-glutamine	-0.2121	0.5563	0.9235
129	carbohydrate binding	SI	Citric acid	-0.2893	0.4176	0.8245
130	carbohydrate binding	SI	A ketoglutaric acid	-0.2121	0.5563	0.9235
131	carbohydrate binding	SI	L-Glutamic acid	0.4062	0.2441	0.8175
132	carbohydrate binding	SI	Isocitric acid	0.4545	0.1869	0.8175
